# Osteonecrosis of the jaw and survival of patients with cancer: a nationwide cohort study in Denmark

**DOI:** 10.1002/cam4.1173

**Published:** 2017-09-21

**Authors:** Priscila Corraini, Uffe Heide‐Jørgensen, Morten Schiødt, Sven Erik Nørholt, John Acquavella, Henrik Toft Sørensen, Vera Ehrenstein

**Affiliations:** ^1^ Department of Clinical Epidemiology Aarhus University Hospital Aarhus N Denmark; ^2^ Department of Oral and Maxillofacial Surgery Rigshospitalet Copenhagen Ø Denmark; ^3^ Department of Oral and Maxillofacial Surgery Aarhus University and Aarhus University Hospital Aarhus Denmark

**Keywords:** Bisphosphonates, bone density conservation agents, drug‐related side effects and adverse reactions, mortality, osteonecrosis

## Abstract

Osteonecrosis of the jaw (ONJ) is an adverse effect of bone‐targeted therapies, which are used to prevent symptomatic skeletal events following bone malignancy. We examined the association between ONJ and survival among cancer patients treated with bone‐targeted agents. Using nationwide registries and databases in Denmark, we identified 184 cancer patients with incident ONJ between 2010 and 2015, and a comparison cohort of 1067 cancer patients without ONJ and with a history of hospital‐administered treatment with bisphosphonates or denosumab initiating from cancer diagnosis. At the date of confirmed ONJ diagnosis, the comparison cohort was matched to the ONJ patients on age, cancer site, year of cancer diagnosis, and stage at diagnosis. The patients were followed up for survival until emigration or 15 June 2016. We computed overall survival and estimated mortality rate ratios adjusted for sex, and for the presence of distant metastases and other comorbidity at start of follow‐up. A match was found for 149 of the 184 ONJ patients. The 1‐ and 3‐year survival among all 184 cancer patients with ONJ was 70% (95% confidence interval [CI]: 63%–76%) and 42% (95% CI: 34%–51%), respectively. Among the matched patients, ONJ was associated with an adjusted mortality rate ratio of 1.31 (95% CI: 1.01–1.71). ONJ was associated with reduced survival among cancer patients treated with bone‐targeted agents. ONJ may be a marker of advanced disease or of survival‐related lifestyle characteristics.

## Introduction

Bone‐targeted agents, such as bisphosphonates or denosumab, are used in patients with bone malignancy to prevent symptomatic skeletal events: pathological fractures, spinal cord compression, or need for radiation or surgery to bone 1, 2. The bone malignancies include multiple myeloma and bone metastases from solid tumors, primarily of prostate, breast, or lung 1. Preventing symptomatic skeletal events improves quality of life. In multiple myeloma and in the adjuvant setting of breast cancer, bone‐targeted therapy may also delay disease progression and prolong survival 3, 4, 5, 6.

Osteonecrosis of the jaw (ONJ), first described in 2003 after intravenous bisphosphonate use 7, is a potentially severe adverse effect of bone‐targeted therapy 8. Among currently or previously treated patients, ONJ is diagnosed in the presence of exposed bone or bone that can be probed through a fistula in the maxillofacial region 8. The lesion must persist for at least 8 weeks in the absence of signs of malignancy or history of radiotherapy to the jaws 8. In clinical trials, up to 5% of cancer patients develop ONJ as late as 3 years of bone‐targeted therapy 9, 10, 11, 12, 13, 14. Treatment of ONJ may involve discontinuation of bone‐targeted agents 15, 16, 17, despite their therapeutic benefits 11, 14.

Data on survival following ONJ among cancer patients will help contribute to the understanding of risk–benefit trade‐offs associated with bone‐targeted therapy for cancer. Previously, survival following ONJ was studied in a cohort of 21 renal cell carcinoma patients treated with zoledronic acid, whereby patients developing ONJ (*N* = 6) had a 17‐month longer median survival compared with patients not developing ONJ 15. Since follow‐up started at cancer diagnosis, which may precede ONJ by years, the observed association could be likely a result of immortal time bias 18.

We conducted a nationwide cohort study in Denmark to examine the association between ONJ and survival among cancer patients treated with bone‐targeted agents.

## Materials and Methods

### Setting

Denmark provides tax‐funded health care to all its residents, and Danish registries and databases contain extensive individual‐level health care data, including data on cancer therapies from 1999 19. Individual‐level data from all data sources are linkable, enabling virtually complete follow‐up 20.

### Cancer population

The study eligibility period started on 15 October 2010, the date when the first ONJ case in a cancer patient was recorded in the Danish ONJ database (described below). We used the Danish Cancer Registry (DCR) and the Danish National Patient Registry (DNPR) to identify all cancer patients who were alive and residing in Denmark between 15 October 2010 and 31 December 2015. The DCR has recorded all incident cancers in Denmark since 1943 21 and is virtually complete 22. Recorded data include diagnosis date, cancer site and stage at diagnosis. Since the DCR was updated until 2013 at the time of this study, we used the DNPR to identify cancer patients diagnosed in 2014–2015. The completeness of the DNPR compared with the DCR is 87–96% 23, 24. The DNPR contains information on all hospitalizations since 1977 and on all outpatient clinic visits since 1995. Diagnoses were registered using the *International Classification of Diseases*,* Eighth Revision* (ICD‐8) through 1993 and the ICD‐10 thereafter. In Denmark, cancer treatments, including denosumab and intravenous bisphosphonates, are administered in hospitals. Intravenous bisphosphonates were introduced to the Danish hospital sector in 1997, 2 years after their approval in the European Union for multiple myeloma and 1 year after their approval for prevention of skeletal events following bone metastasis. Denosumab was introduced for skeletal events prevention in 2011 25.

### The ONJ cohort

The ONJ cohort consisted of patients from the cancer population with incident clinically confirmed ONJ, as recorded in the Danish ONJ database. This research database was established to register ONJ events nationwide 26. In Denmark, all patients with suspected ONJ are referred to hospital‐based departments of oral and maxillofacial surgery 26. Specialists in these departments (physicians, dentists, or oral surgeons) report the confirmed ONJ diagnoses to the Danish ONJ database. The database was established in October 2011; confirmed ONJs diagnosed between October 2010 and September 2011 were registered retrospectively using information from medical records.

### The comparison cohort

We used the DNPR and the Danish ONJ database to construct a comparison cohort of cancer patients without ONJ and with a record of hospital‐administered treatment with bisphosphonates or denosumab. Cancer patients receiving treatment exclusively for osteoporosis have a lower ONJ risk 27 and presumably a better prognosis. Therefore, we selected only treatment records initiating from cancer diagnosis date and before the date of confirmed ONJ diagnosis (defined as the index date). For each ONJ patient, we randomly sampled up to 10 patients from the treated cancer population who were alive and without ONJ on the index date, matched on birth year (within 3 years), cancer site, year of cancer diagnosis, and distant stage at cancer diagnosis. Patients with records of diagnostic codes indicative of ONJ in the DNPR before the index date were not eligible to enter the comparison cohort 28.

### Covariates

From the DNPR, we obtained information on recorded diagnoses of distant metastases at the index date, and on the comorbidity level, since 1977, expressed using the Charlson Comorbidity Index score (excluding cancer), as 0, 1–2, or 3+ 29, 30.

Table S1 lists definitions of the study variables.

### Statistical analysis

Patients were followed from the index date until the earliest of death, emigration, or 15 June 2016. We used the Kaplan–Meier estimator to compute and display survival both for the ONJ patients and the comparison cohort. Furthermore, we constructed adjusted survival curves using stabilized weights that represented each patient's inverse probability of having ONJ. Briefly, these weights were estimated in a logistic regression model with ONJ as the dependent variable, and sex, distant metastasis, and comorbidity level on the index date as the independent variables 31. We used stratified Cox regression to compute hazard ratios as estimates of mortality rate ratios (MRRs) and the associated 95% confidence intervals (CIs) adjusted for sex, and for the presence of distant metastasis, and the level of other comorbidity on the index date. The proportionality assumption was examined using Kolmogorov‐type supremum tests 32.

To assess the impact of potential incomplete registration of ONJ before 2012, we performed a sensitivity analysis restricted to patients enrolled between 2012 and 2015. Finally, we performed an additional sensitivity analysis restricted to patients without a diagnosis of osteoporosis. Analyses were conducted using SAS software version 9.4 (SAS Inc., Cary, NC). The study was approved by the Danish Data Protection Agency (record number 2012‐41‐0045). Patient consent is not required by Danish law for studies based on routine electronic data.

## Results

During the study period, we identified 184 patients for inclusion in the ONJ cohort and 1067 patients for inclusion in the comparison cohort (Table 1). Eighty‐nine percent of patients had breast cancer or prostate cancer. Median age at index date was 68 years (interquartile range [IQR]: 63–74 years). Median time from cancer diagnosis to confirmed ONJ was 6.3 years (IQR: 2.9–11 years). Thirty‐five patients in the ONJ cohort (80% of whom had cancers at sites other than breast and prostate) could not be matched to anyone with a history of bone‐targeted therapy, resulting in a matched ONJ cohort of 149 patients (Table 1). The median time from first‐recorded therapy to the index date was 2.1 years (IQR: 1.2–3.6 years) in the matched ONJ cohort, and 1.4 years in the comparison cohort (IQR: 0.6–2.9 years).

**Table 1:** Characteristics of the osteonecrosis of the jaw (ONJ) cohort and the comparison cohort

Characteristics	ONJ cohort (*N* = 184)		Matched ONJ cohort (*N* = 149)		Comparison cohort (*N* = 1067)	
	*N*	%	*N*	%	*N*	%
Age on the index date (years)						
36–54	18	9.78	14	9.40	69	6.47
55–64	44	23.9	38	25.5	261	24.5
65–79	97	52.7	82	55.0	679	63.6
80–94	25	13.6	15	10.1	58	5.44
Sex						
Female	115	62.5	96	64.4	687	64.4
Index year						
2010–2011	9	4.89	6	4.03	30	2.81
2012–2015	175	95.1	143	96.0	1037	97.2
Year of cancer diagnosis						
1980–1994	17	9.24	10	6.71	72	6.75
1995–1999	14	7.61	12	8.05	93	8.72
2000–2003	22	12.0	16	10.7	129	12.1
2004–2013	124	67.4	105	70.5	728	68.2
2014–2015	7	3.80	6	4.03	45	4.22
Cancer site						
Breast	89	48.4	84	56.4	645	60.4
Prostate	46	25.0	44	29.5	331	31.0
Kidney	15	8.15	4	2.68	4	0.37
Multiple myeloma	9	4.89	8	5.37	62	5.81
Lung	8	4.35	6	4.03	20	1.87
Other cancer sites	17	9.24	3	2.01	5	0.47
Cancer stage at diagnosis						
Local or regional	98	53.3	81	54.4	670	62.8
Distant metastasis	47	25.5	38	25.5	200	18.7
Unknown stage	27	14.7	22	14.8	135	12.7
N/A (nonsolid tumors)	12	6.52	8	5.37	62	5.81
Distant metastasis on the index date	111	60.3	91	61.1	470	44.1
Years from cancer diagnosis to the index date*,* median (IQR)	6.3 (2.9–11)		6.1 (2.9–9.9)		6.6 (3.2–10)	
History of hospital‐based therapy with bone‐targeted agents	158	85.9	141	94.6	1067	100
History of osteoporosis	25	13.6	14	9.40	186	17.4
Charlson Comorbidity Index score^a^						
0	99	53.8	87	58.4	706	66.2
1–2	69	37.5	48	32.2	295	27.7
3+	16	8.70	14	9.40	66	6.19

Other cancer sites included other primary and secondary cancers. IQR, interquartile range; *N*, number of individuals; Comparison cohort, cohort within the cancer population without ONJ and with a recorded history of hospital‐based therapy with bone‐targeted agents starting from cancer diagnosis date; N/A, nonapplicable.

a Excluding cancer.

**Figure 1:**
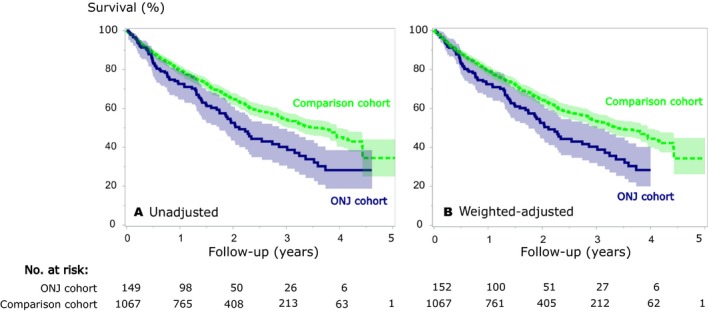
Overall survival and corresponding 95% CIs (shaded areas) in the osteonecrosis of the jaw (ONJ,* N* = 149) and in the matched comparison cohort (*N* = 1067). (A) Unadjusted survival curves and (B) adjusted survival curves using inverse probability weights. The ONJ confirmation date served as the index date for the ONJ cohort and matched members of the comparison cohort.

During median follow‐up of 1.4 years (IQR: 0.7–2.3 years) in the ONJ cohort, 1‐year survival was 70% (95% CI: 63%–76%) and 3‐year survival was 42% (95% CI: 34%–51%). One‐year survival in the ONJ cohort was 77% (95% CI: 67%–85%) among breast cancer patients and 66% (95% CI: 50%–78%) among prostate cancer patients. Three‐year survival was 47% (95% CI: 35%–58%) among breast cancer patients with ONJ and 29% (95% CI: 13%–47%) among prostate cancer patients with ONJ. The comparison cohort had a median follow‐up of 1.8 years (IQR: 0.9–2.6 years), and the 1‐ and 3‐year survival estimates were 80% (95% CI: 77%–82%) and 54% (95% CI: 50%–58%), respectively (Fig. 1A).

Among the matched patients, ONJ was associated with an adjusted MRR of 1.31 (95% CI: 1.01–1.71) (Table 2 and Fig. 1B). The estimated median survival time was 2.1 years in the ONJ cohort and 3.4 years in the matched comparison cohort. Although imprecisely measured, the MRR was higher for prostate cancer than for breast cancer patients (Table 2). The sensitivity analyses restricted to the 2012–2015 eligibility period or to cancer patients without a diagnosis of osteoporosis produced consistent results compared with the main analysis (Table S2).

**Table 2:** All‐cause mortality rates and mortality rate ratios (MRRs) in cancer patients with osteonecrosis of the jaw (ONJ), relative to the comparison cohort

Cancer site	Cohort	*N*	*N* deaths	Person‐years	All‐cause mortality					
					Mortality rate		Mortality rate ratios			
					Rate^a^	95% CI	Unadjusted	95% CI	Adjusted^b^	95% CI
All	ONJ	149	80	253	31.6	25.0–39.3	1.48	1.14–1.92	1.31	1.01–1.71
	Comparison	1067	417	1994	20.9	19.0–23.0	Reference	—	Reference	—
Breast	ONJ	84	42	165	25.5	18.4–34.5	1.34	0.95–1.90	1.13	0.80–1.60
	Comparison	645	250	1276	19.6	17.2–22.2	Reference	—	Reference	—
Prostate	ONJ	44	26	60	43.4	28.4–63.6	1.72	1.08–2.74	1.60	0.99–2.58
	Comparison	331	127	540	23.5	19.6–28.0	Reference	—	Reference	—
Other	ONJ	21	12	29	41.7	21.5–72.8	c		c	
	Comparison	91	40	178	22.4	16.0–30.5				

Stratified analyses by cancer site are provided according to: breast cancer, prostate cancer and other cancer sites. *N*, number of individuals; Comparison cohort, comparison cohort from the cancer population without ONJ and with a record of bone‐targeted therapy initiating from cancer diagnosis date.

a Per 100 person‐years.

b Adjusted for record of distant metastasis (yes/no) registered on/before the index date, level of other comorbidity, and for sex (except in analyses for prostate cancer and for breast cancer).

c Proportionality assumption was not fulfilled, as verified using Kolmogorov‐type supremum tests (unadjusted model, *P* = 0.014; adjusted model, *P* = 0.010).

## Discussion

In this cohort study of cancer patients treated with bone‐targeted agents, 1‐year survival after incident ONJ was 70%. Less than half of the ONJ‐affected patients survived for 3 years. After adjustment for measured prognostic factors, ONJ was associated with a mortality rate ratio of 1.31 compared with patients without ONJ. This finding contradicts the previously reported prolonged survival associated with ONJ 15.

We report results from the largest nationwide cohort of cancer patients to date with clinically confirmed ONJ, with complete follow‐up for death and migrations. Although ascertainment of ONJ was potentially incomplete before 2012, our results did not materially change after restriction to a period with more complete ONJ ascertainment. Among cancer patients, the positive predictive value of a DNPR‐recorded hospital administration of intravenous bisphosphonate is 98% 33. However, the indication for bone‐targeted therapies is not recorded in the DNPR. Although the prevalence of osteoporosis was higher in the comparison cohort (17%) than in the ONJ cohort (9%), excluding patients with osteoporosis did not affect our MRRs. A smaller proportion of cancer patients without osteoporosis could nevertheless have received bone‐targeted agents to prevent cancer treatment‐induced bone loss 1, 34. It is not known whether cancer therapy‐induced bone loss affects survival aside from its associated cancer stage. Therefore, we have not considered it as a potential confounder in our study.

The association of ONJ with mortality remaining after adjustment for measured prognostic factors may be due to ONJ being a marker of underlying advanced disease, or of behaviors associated with survival, such as smoking 35, 36. Missing data on cancer stage at diagnosis was observed in nearly 13–15% of the cohorts. Subsequently, missing registration on distant cancer stage is expected to be higher during the median period of 6 years from cancer diagnosis until the index date. Nonetheless, assuming most cancer patients received bone‐targeted therapy for bone malignancy 33, 34, and that time elapsed from first therapy record to the index date was longer in the ONJ than in the comparison cohort, ONJ patients could have more advanced bone disease at start follow‐up than members of the comparison cohort.

In conclusion, ONJ was associated with reduced survival among cancer patients treated with bone‐targeted agents. ONJ may be a marker of advanced disease or of survival‐related lifestyle characteristics.

## Conflict of Interest

The authors have declared no conflicts of interest.

## Supporting information


**Table S1.** Definitions of study variables by their specific ICD and procedure codes.
**Table S2.** Sensitivity analyses of the association between ONJ and mortality among cancer patients treated with bone‐targeted agents, restricting the study eligibility period to 2012–2015, or to patients without osteoporosis.
