# Supplementary material for: Osteonecrosis of the jaw and survival of patients with cancer: a nationwide cohort study in Denmark
Source: Cancer Med. 2017 Sep 21;6(10):2271–7. doi: 10.1002/cam4.1173 (PMC5633555; doi:10.1002/cam4.1173)
Supplement: Supplementary file 1 — Table S1. Definitions of study variables by their specific ICD and procedure codes. Table S2. Sensitivity analyses of the association between ONJ and mortality among cancer patients treated with bone‐targeted agents, restricting the study eligibility period to 2012–2015, or to patients without osteoporosis. [file CAM4-6-2271-s001.docx]

**Online Supporting Information**

Osteonecrosis of the jaw and survival of patients with cancer: A nationwide cohort study in Denmark

**Supporting Table 1.** Definitions of study variables by their specific ICD and procedure codes.

| **Disease or condition** | **ICD-8** | | **ICD-10** |
| --- | --- | --- | --- |
| **Primary Cancer Site** |  | | **C00-C96** |
| *Subtypes of interest:* |  | |  |
| Multiple myeloma |  | | C90 |
| Breast |  | | C50 |
| Prostate |  | | C61 |
| Kidney |  | | C64 |
| Lung |  | | C34 |
| Others  (including secondary cancers) |  | | C15-C29, C33, C35-C43 C45-C49, C51-C60, C62, C63, C65-C89, C91-C96 |
| **Distant metastasis stage** | 195-199 | | C76-C80  From 2003-: **TNM classification^a^** |
| **Comorbidity included in the CCI**^b^ | **ICD-8** | | **ICD-10** |
| Myocardial infarction | 410 | | I21-I23 |
| Congestive heart failure | 427.09-427.11, 427.19, 428.99, 782.49 | | I50, I11.0, I13.0, I13.2 |
| Peripheral vascular disease  Cerebrovascular disease | 440-445  430-434, 436-438 | | I70-I74, I77  I60-I69, G45, G46 |
| Dementia | 290.09-290.19, 293.09 | | F00-F03, F05.1, G30 |
| Chronic pulmonary disease | 490-493, 515-518 | | J40-J47, J60-J67, J68.4, J70.1 |
| Connective tissue disease | 712, 716, 734, 446, 135.99 | | M05, M06, M08, M09, M30, M31, M32, M33, M34, M35, M36, D86 |
| Ulcer disease | 530.91, 530.98, 531-534 | | K22.1, K25-K28 |
| Mild liver disease | 571, 573.01, 573.04 | | B18, K70.0-K70.3, K70.9, K71, K73, K74, K76.0 |
| Diabetes types I & II | 249.00, 249.06, 249.07, 249.09, 250.00, 250.06, 250.07, 250.09 | | E10.0, E10.1, E10.9, E11.0, E11.1, E11.9 |
| Hemiplegia | 344 | | G81, G82 |
| Moderate to severe renal disease | 403, 404, 580-583, 584, 590.09, 593.19, 753.10-753.19, 792 | | I12, I13, N00-N05, N07, N11, N14, N17-N19, Q61 |
| Diabetes with end-organ damage | 249.01-249.05, 249.08, 250.01-250.05, 250.08 | | E10.2-E10.8, E11.2-E11.8 |
| Moderate to severe liver disease | 070.00, 070.02, 070.04, 070.06, 070.08, 573.00, 456.00-456.09 | | B15.0, B16.0, B16.2, B19.0, K70.4, K72, K76.6, I85; |
| AIDS | 079.83 | | B21-B24 |
| **Osteoporosis** | | M80-M82 | |
| **Hospital-based therapy with bone-targeted agents** | | **procedure code** | |
| Bisphosphonates | BWHB40 | | |
| Denosumab | BWHB42 | | |
| **Exclusion criteria – comparison cohort** | | | **ICD-10** |
| Inflammatory conditions of the jaw |  | | K10.2 |
| Osteonecrosis |  | | M87.0-87.9 |
| Alveolitis of the jaws |  | | K10.3 |
| Periapical abscess with sinus |  | | K04.6 |

CCI: Charlson Comorbidity Index

^a^The TNM criteria for distant metastasis according to different types of primary tumors can be found at: Sobin LH, Gospodarowicz MK, Wittekind C, eds. TNM Classification of Malignant Tumors (7^th^ edition). Hoboken, NJ, USA: Wiley-Blackwell, 2009.

^b^Except for cancer

**Supporting Table 2.** Sensitivity analyses of the association between ONJ and mortality among cancer patients treated with bone-targeted agents, restricting the study eligibility period to 2012-2015, or to patients without osteoporosis.

| **Analysis**  **restricted to** |  |  | |  | |  | |  | | **All-cause mortality** | | | | | | | |
| --- | --- | --- | --- | --- | --- | --- | --- | --- | --- | --- | --- | --- | --- | --- | --- | --- | --- |
|  | **Cohort** | **N** | **N deaths** | | **Person-years** | | **Mortality rate** | | | |  | |  | **Mortality rate ratios** | | | |
|  |  |  |  |  |  |  | **Rate^a^** | | **95% CI** | | **Unadjusted** | | | | **95% CI** | **Adjusted^b^** | **95% CI** |
| Patients eligible in 2012-2015 | ONJ | 143 | 77 | | 233 | | 33.1 | | 26.1-41.4 | | | 1.56 | | | 1.20-2.04 | 1.40 | 1.07-1.83 |
|  | Comparison | 1,037 | 398 | | 1,898 | | 21.0 | | 19.0-23.1 | | | reference | | | - | reference | *-* |
| Cancer patients without osteoporosis | ONJ | 135 | 78 | | 220 | | 35.5 | | 28.0-44.3 | | | 1.50 | | | 1.14-1.97 | 1.32 | 1.00-1.74 |
|  | Comparison | 881 | 378 | | 1,610 | | 23.5 | | 21.2-26.0 | | | reference | | | - | reference | - |

^a^Per 100 person-years

^b^Adjusted by presence of distant metastases registered on/before the index date, level of other comorbidity, and sex.

Abbreviations: N = number of individuals, ONJ = osteonecrosis of the jaw; Comparison cohort = comparison cohort from the cancer population without ONJ and with a record of bone-targeted therapy starting from cancer diagnosis date.
